# Clicking on *trans*-translation drug targets

**DOI:** 10.3389/fmicb.2015.00498

**Published:** 2015-05-19

**Authors:** John N. Alumasa, Kenneth C. Keiler

**Affiliations:** Department of Biochemistry and Molecular Biology, Pennsylvania State UniversityUniversity Park, PA, USA

**Keywords:** antibacterial, *trans*-translation, molecular target identification, chemical biology, click chemistry, photo-affinity labeling, fluorescent probe

## Introduction

The increase in drug resistant bacteria continues to pose a significant threat to human health worldwide, and new antibiotics are required to combat resistant strains (Center for disease Control and Prevention, 2013). Drug discovery typically begins with identification of an appropriate target pathway, isolation of “hit” compounds that inhibit (or dysregulate) the pathway, validation, and optimization of the hit into a lead compound for development. *trans*-Translation has been an intriguing target for new antibiotics because the pathway is required for virulence or viability in most bacterial pathogens that have been examined, but it is not present in metazoans, so specific inhibitors might have few side effects (Keiler and Alumasa, 2013; Ramadoss et al., 2013; Giudice et al., 2014). Recent publication of a high-throughput screen capable of identifying inhibitors of *trans*-translation with antibiotic activity validated the pathway as a potential drug target and provided a key tool for isolating lead compounds (Ramadoss et al., 2013). We argue that this high-throughput assay, coupled to click-chemistry techniques to identify the molecular targets of active compounds, should enable widespread efforts to develop drugs targeted to *trans*-translation.

## Reasons for targeting *trans*-translation

*trans*-Translation is a pathway for rescuing ribosomes trapped at the 3' end of an mRNA (Keiler et al., 1996). Bacteria do not use the same mRNA quality control mechanisms employed by eukaryotes to ensure that mRNAs are intact before translation begins (Keiler, 2008). As a result, translation can initiate on truncated or damaged mRNAs. In addition, mRNA damage can occur after ribosomes have initiated translation. When the ribosome reaches the 3′ end of such an mRNA, there is no stop codon to terminate translation. Estimates in *E. coli* indicate that ~2–4% of all translation initiations terminate in stalled complexes (Ito et al., 2011). Accumulation of these stalled complexes is lethal, and bacteria require at least one mechanism to rescue stalled ribosomes (Feaga et al., 2014; Keiler and Feaga, 2014). The primary rescue pathway in bacteria is *trans*-translation, in which the protein SmpB and the specialized RNA, tmRNA, recognize stalled ribosomes. During *trans*-translation, the ribosome resumes translation on an open reading frame within tmRNA, adding a peptide tag to the C terminus of the nascent polypeptide and releasing the previously stalled ribosome. The peptide tag is ultimately recognized by multiple proteases, which degrade the tagged protein (Keiler, 2008). Genes encoding tmRNA and SmpB have been identified in almost all bacterial genomes (Hudson et al., 2014). Some species also have backup rescue systems, the ArfA or ArfB proteins (Chadani et al., 2011, 2012) that can release ribosomes when *trans*-translation activity is not available.

A number of key attributes make *trans*-translation an attractive antibiotic target. First, it is essential for viability or virulence in many pathogenic species, including *Neisseria gonorrhoeae*, *Mycobacterium tuberculosis*, and *Staphylococcus aureus* (Huang et al., 2000; Zhang et al., 2012b; Fey et al., 2013). Disruption of *trans*-translation also results in hyper-sensitivity to antibiotics in some species (Abo et al., 2002; Li et al., 2013). Second, tmRNA and SmpB are universally conserved in bacteria, but are absent in metazoans, so specific inhibition of *trans*-translation should not cause toxicity in the host (Hudson et al., 2014). Third, the pathway includes several molecules that are not targeted by existing antibiotics, including tmRNA, SmpB, and stalled ribosomes, therefore cross-resistance with existing antibiotics should be minimal.

## An HTS assay to identify inhibitors

To validate *trans*-translation as a target and to begin the drug discovery process, an assay for *trans*-translation inhibitors was developed and used in a high-throughput screen (Ramadoss et al., 2013). The assay uses an *E. coli* strain engineered to express luciferase from a truncated mRNA. When there is no inhibition, all the luciferase is tagged by *trans*-translation and degraded, but when *trans*-translation is inhibited active luciferase is produced. The Z' score under high-throughput conditions is 0.75, and the assay is appropriate for a wide range of screening platforms. Screening of a library of small molecules identified specific inhibitors that have promising antibacterial properties against both Gram-positive and Gram-negative bacteria and exhibit low toxicity against human cells (Ramadoss et al., 2013).

This assay is cell-based, which ensures that hit compounds will be able to function in the context of the bacterial cell. However, inhibitors could target one of several potential molecules including both nucleic acids and proteins (Keiler and Alumasa, 2013; Ramadoss et al., 2013; Giudice et al., 2014), and the molecular target of each inhibitor must be identified to facilitate optimization.

## Molecular target identification strategies

The main genetic approach to molecular target identification typically involves isolating and sequencing resistant mutants to identify candidate targets (Lerner et al., 2005; Wacker et al., 2012; Schenone et al., 2013). This method is generally successful when resistant mutants can be obtained (Wacker et al., 2012). However, there are several reasons why target-based resistance will be rare or nonexistent: (i) mutations in the target that eliminate inhibitor activity may themselves be lethal; (ii) multiple mutations may be required for resistance, for example if the target is encoded in redundant genes; (iii) there may be multiple targets for the compound. No target-based resistant mutants have been obtained for any of the *trans*-translation inhibitors, although it is not known why resistance is rare in this case (Ramadoss et al., 2013).

Alternative approaches to identify molecular targets use chemical biology, computational, or biochemical techniques (Schenone et al., 2013). Chemical biology techniques typically employ the use of small molecule probes that mimic the structure of the inhibitor. Probe-aided pull down assays, which use an affinity tag (biotin) fused to the inhibitor, fall into this category. Although this approach has been shown to work independently, greater success occurs when it is coupled with orthogonal methods such as photo-affinity labeling (Li et al., 2004; Kotake et al., 2007; Yamazaki et al., 2010). A significant drawback of this approach is that structural modifications performed on the pharmacophore to incorporate the affinity tag and a linker usually result in altered physicochemical properties of the probe that may affect target binding (Kashiwayama et al., 2010; Zhang et al., 2012a). Click chemistry could provide a solution to this problem through a two step process incorporating the use of a minimally-modified pharmacophore for target binding, and a secondary probe for isolation of the target molecule.

## Click chemistry

**Figure 1:**
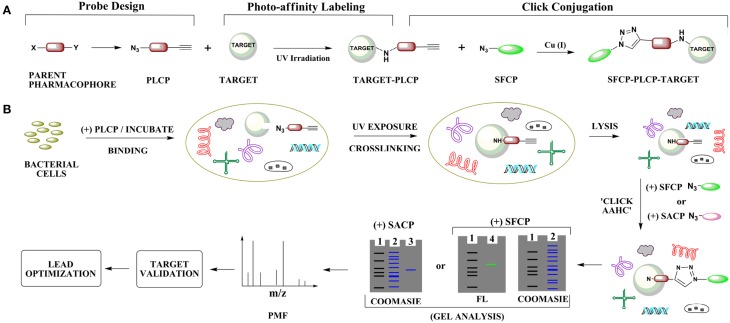
**Identification of molecular targets by photo-affinity labeling and click chemistry. (A)** Overview of photo-labile click probe (PLCP) design, photo-affinity labeling and click bioconjugation using a fluorescent reporter probe. **(B)** Cell-based strategy for click-based molecular target identification for *trans*-translation inhibitors. The process involves intracellular probe-target binding, intracellular probe-target covalent cross-linking, cell lysis, click bioconjugation using either a secondary fluorescent click probe (SFCP) or secondary affinity click probe (SACP), target isolation, identification by peptide mass fingerprinting (PMF), and validation all leading to lead optimization. Abbreviations: AAHC, Azide-alkyne Huisgen Cycloaddition; FL, fluorescence-exposed gel; Gel lanes, one ladder; two cell lysate; three target isolated from a click-based affinity pull-down assay; 4 target isolated from a click-based fluorescence assay.

The concept of click chemistry broadly encompasses groups of chemical reactions that are fast, versatile, high yielding and simple to use (Kolb et al., 2001). The copper catalyzed Azide-Alkyne Huisgen Cycloaddition (AAHC) is among the most common click chemistry reactions (Spiteri and Moses, 2010). AAHC requires both azide and alkyne functional groups, which react almost exclusively with each other under a defined set of conditions, minimizing unwanted side reactions (Thirumurugan et al., 2013). Notable advantages of this method include its specificity, mild reaction conditions, irreversibility and selectivity (Kolb and Sharpless, 2003). When applied to molecular target identification, AAHC can be coupled with methods such as photo-affinity labeling, making the identification process more efficient (Sumranjit and Chung, 2013). In the example we consider here, photo-affinity labeling can be used to covalently attach a probe to the target, and a subsequent AAHC reaction can link the probe to a fluorescent moiety or affinity tag to enable isolation (Figure 1). This strategy circumvents addition of bulky substituents to the probe that may otherwise affect probe-target binding.

Compatibility of this reaction with physiological conditions provides myriad avenues for target identification studies, and this procedure has been successfully employed to identify molecular targets for anticancer and antibacterial agents. For example, the click chemistry approach was used to identify the target for LW6 an inhibitor targeting the regulation of tumor angiogenesis and metastasis in colon cancer cell lines (Lee et al., 2013). Similarly, this procedure was successfully used to identify inhibitors of the biotin protein ligase possessing antibacterial activity (Tieu et al., 2013). Many other examples have been described, demonstrating the efficiency of this method in identifying molecular targets within biological environments (Colca et al., 2003; Thirumurugan et al., 2013). It is noteworthy that the click-based method can be used in live cells, in contrast to pull-down assays that are normally performed *in vitro* (Colca et al., 2003; Speers and Cravatt, 2004). This is an important property that would allow probe-target interaction to occur in cells, prior to target isolation. For all such techniques, however, success is largely dependent upon nature of the probe.

## Click probe design

Probe design is perhaps the most challenging task requiring careful assessment in the initial steps of target identification. An efficient click probe must retain key properties of the parent molecule, including biological activity and cell permeability, while accommodating the azide and alkyne moieties that enable target cross-linking and subsequent click coupling. Placement of these groups can be guided by structure-activity relationship (SAR) studies aimed at identifying sites within the basic pharmacophore that allow modification without eliminating activity or increasing off-target binding interactions. A generalized design for a photo-labile click probe (PLCP), harboring a photo-labile terminal azide group and a terminal alkyne, is shown in Figure 1A. The azide group in this PLCP can be activated by UV irradiation to cross-link the PLCP to the target. The alkyne can then be used for click conjugation to a secondary probe containing a fluorescent or biotinylated group to facilitate purification.

## Molecular target identification

To identify the molecular target, the PLCP can be cross-linked to interacting molecules *in vivo*, in a cell extract, or in a purified system. If the probe is cell permeable, it can be added to bacterial cells to allow target binding (Figure 1B) (Colca et al., 2003). UV irradiation (typically 254–366 nm) is then used to activate the azide and cross-link the PCLP to binding partners. The cells are lysed under denaturing conditions, excess PCLP is removed, and the secondary probe is attached via click chemistry. Denaturation ensures that the click reaction can proceed even if the PCLP binds in a site that is inaccessible under native conditions. If a fluorescent secondary probe is attached, gel electrophoresis can be used to isolate fluorescent bands (Figure 1B). Conversely, if the secondary probe contains biotin or any other affinity tag, the target can be isolated on an appropriate affinity resin (Pozdnyalov et al., 2013). In either case, the target molecule is then identified by mass spectrometry. If the PLCP is not cell permeable, it can be added to a cell extract or to purified candidate molecules prior to UV irradiation, but the rest of the identification procedure would be unchanged. Once the target is identified, target validation and lead optimization can proceed.

## Summary

*trans*-Translation is an attractive target for new antibiotic development because the reaction is required for virulence or viability in many pathogens, and several small molecules that inhibit the pathway have been identified (Ramadoss et al., 2013). A cell-based assay for high-throughput screening has been engineered, enabling screening of diverse libraries for inhibitors that are active *in vivo* (Ramadoss et al., 2013). The click-based molecular target identification strategy described here should enable rapid advancement through the hit validation process. With these tools available, *trans*-translation should be pursued as a drug target on many fronts.

### Conflict of interest statement

The authors declare that the research was conducted in the absence of any commercial or financial relationships that could be construed as a potential conflict of interest.
